# Questionnaire on Nursing Competencies in Nutritional Care for Chronic Kidney Patients: Development and Validation

**DOI:** 10.3390/nursrep16030078

**Published:** 2026-02-24

**Authors:** Gaetano Ferrara, Mattia Bozzetti, Marco Sguanci, Loris Bonetti, Sara Morales Palomares, Elena Sandri, Giovanni Cangelosi, Daniele Napolitano, Italian Society of Nephrology Nurse (SIAN) Research Group, Stefano Mancin, Michela Piredda

**Affiliations:** 1 Department of Biomedicine and Prevention, University of Rome Tor Vergata, Viale Montpellier 1, 00128 Rome, Italy; gaetano.ferrara@students.uniroma2.eu; 2 Italian Society of Nephrology Nurse (SIAN), Via Capotesta 1/30, 07026 Olbia, Italy Research Group);; 3 Direction of Health Professions, ASST Cremona, Viale Concordia 1, 26100 Cremona, Italy; 4 Nursing Research Competence Centre, Ente Ospedaliero Cantonale, 3 Officina Avenue, 6500 Bellinzona, Switzerland; 5 Department of Business Economics, Health and Social Care, University of Applied Sciences and Arts of Southern Switzerland, Building E, 16e Cantonale Street, 6928 Manno, Switzerland; 6 Department of Pharmacy, Health and Nutritional Sciences (DFSSN), University of Calabria, 87036 Rende, Italy; 7 Faculty of Medicine and Health Sciences, Catholic University of Valencia San Vicente Martir, 46001 Valencia, Spain; 8 School of Pharmacy, Experimental Medicine and “Stefania Scuri” Public Health Department, University of Camerino, Via Madonna delle Carceri 9, 62032 Camerino, Italy; 9 CEMAD, Fondazione Policlinico Gemelli IRCCS, Largo Agostino Gemelli 1, 00168 Rome, Italy; daniele.napolitano@policlinicogemelli.it; 10 Research Unit Nursing Science, Department of Medicine and Surgery, Campus Bio-Medico di Roma University, Via Álvaro del Portillo 21, 00128 Rome, Italy; m.piredda@unicampus.it

**Keywords:** nephrology nursing, nutritional care, chronic kidney disease, competency assessment, psychometrics validation

## Abstract

**Background/Objectives**: Nutritional management is central to the care of patients with end-stage renal disease (ESRD), yet malnutrition often remains under-recognized due to gaps in nursing knowledge and competencies. This study aimed to develop and validate the Nursing Education and Competencies in Nutrition for Patients with CKD in ESRD (NECN-ESRD) questionnaire, designed to assess nephrology nurses’ competencies, attitudes, and practices in nutritional care. **Methods**: A methodological and cross-sectional design was adopted, following the COnsensus-based Standards for the selection of health Measurement INstruments (COSMIN) recommendations for instrument development. The process comprised five phases: construct definition and item generation, expert consultation and revision, quantitative content validity analysis, pilot testing, and psychometric testing. Data were collected between August and September 2025 from 405 nephrology nurses across Italy. Exploratory Factor Analyses (EFAs) and Confirmatory Factor Analyses (CFAs) were conducted on split samples (60/40), and key psychometric properties were evaluated. **Results**: EFA identified a four-factor structure—Recommendations, Attitudes, Practice, and Advanced Competencies—which was confirmed through CFA with good fit indices [Comparative Fit Index (CFI) = 0.995, Tucker–Lewis Index (TLI) = 0.994, Root Mean Square Error of Approximation (RMSEA) = 0.07]. A higher-order model further improved fit (CFI = 0.994, RMSEA = 0.029), explaining 68.2% of variance. Internal consistency was excellent (ω = 0.89–0.96), test–retest reliability showed perfect agreement [Intraclass Correlation Coefficient (ICC) = 1.00], and invariance testing supported equivalence across educational and experience levels. **Conclusions**: The NECN-ESRD demonstrated strong validity, reliability, and stability, providing a robust and context-specific tool to assess and enhance nurses’ competencies in nutritional care for ESRD patients. Its application can support targeted educational interventions, improve clinical practice, and contribute to enhancing the quality of nutritional care for patients with ESRD within healthcare systems.

## 1. Introduction

Chronic kidney disease (CKD) affects an estimated 670 million individuals globally, representing a substantial public health burden and ranking among the leading causes of morbidity and mortality worldwide [1]. In Italy, although epidemiological surveillance is limited, current estimates suggest a CKD prevalence of roughly 6–7% in the adult population; this figure is supported by data from the national health examination survey, which reported a 7.05% prevalence among individuals aged 35–79 years [2,3,4]. Nutritional management is a core component of care for individuals living with chronic kidney disease (CKD), including those with end-stage renal disease (ESRD) [5]. The 2020 update of the Kidney Disease Outcomes Quality Initiative (KDOQI) Clinical Practice Guideline for Nutrition in CKD highlights medical nutrition therapy, encompassing assessment and regulation of protein and energy intake, micronutrients, and electrolytes, as integral to slowing disease progression, preventing complications, and improving patient-reported outcomes across CKD stages and dialysis modalities [5,6]. Among nutrition-related complications, protein–energy wasting (PEW) is particularly consequential [7]. The International Society of Renal Nutrition and Metabolism (ISRNM) defines PEW as a pathological state characterized by the depletion of body protein and energy reserves in CKD, distinct from cachexia and associated with increased morbidity and mortality [7,8]. Malnutrition is highly prevalent in CKD and remains a strong predictor of adverse outcomes, particularly among dialysis patients [9]. Malnutrition in CKD is multifactorial, arising from the combined effects of reduced dietary intake, chronic inflammation, hormonal and metabolic disturbances, and the cumulative burden of comorbidities and treatment-related factors [10]. Symptoms such as anorexia, nausea, gastrointestinal disturbances and sensory alterations, common in several chronic diseases, in older adults and in patients with ESRD can further decrease appetite and enjoyment of food, thus exacerbating nutritional decline [11,12,13]. Within multidisciplinary renal teams, nurses play a pivotal role in translating nutrition guidelines into everyday practice. Beyond task-oriented responsibilities, nephrology nurses provide continuous screening and assessment, patient and family education, behavioural counselling, and care coordination, all of which influence dietary adherence, symptom control, and quality of life [14,15,16]. However, knowledge and skill gaps, for instance those related to mineral metabolism and phosphorus management, have been documented among both patients and healthcare providers, underscoring the need for structured competency development in clinical nutrition [17]. Despite the availability of validated tools and clinical guidance, malnutrition frequently remains under-recognized in routine care, partly due to insufficient knowledge and suboptimal attitudes among nurses and other professionals [18,19]. Negative or dismissive attitudes toward malnutrition are associated with failure to identify at-risk patients and delays in initiating appropriate nutritional interventions [20]. Given the prognostic significance of malnutrition in CKD, including its association with long-term mortality even within multidisciplinary care pathways [21], enhancing nurses’ competence, confidence, and practice behaviours in nutritional care is both a clinical and organizational priority. In the nursing context, competence is described as a multidimensional construct encompassing knowledge, skills, capability, and professional attributes required to deliver safe and high-quality nursing care and to support optimal patient outcomes [22]. Higher levels of nursing competence in nutritional care may be associated with earlier identification of malnutrition, improved adherence to nutritional guidelines, better symptom management, and improved patient-reported outcomes. Competency-based education frameworks issued by major nursing bodies [e.g., International Council of Nurses (ICN), American Association of Colleges of Nursing (AACN)] emphasize the explicit definition, assessment, and progression of professional competencies, including those related to clinical nutrition, across all practice levels and settings [23,24,25]. Nurse-led, nutrition-focused programs have demonstrated improvements in knowledge, adherence, and selected patient-reported outcomes; however, their scalability and impact on clinical endpoints depend on standardized training and consistent evaluation [26,27]. Recent international studies have addressed nursing competencies in CKD management. For example, Wei et al. developed a competency evaluation index system for nurses working in chronic kidney disease management centres using expert consensus methods [28]. However, these approaches focus on role delineation and training frameworks rather than on psychometrically validated instruments specifically designed to assess nurses’ nutritional competencies in patients with ESRD. Despite the centrality of nutritional care in ESRD, there is a lack of ESRD-specific, psychometrically validated instruments to measure nephrology nurses’ competencies, attitudes, and practices in nutrition. Robust measurement is essential to identify learning needs, design targeted educational interventions and evaluate their effects on care processes and patient outcomes. The Nursing Education and Competencies in Nutrition for Patients with CKD in ESRD (NECN-ESRD) is a context-specific, theoretically grounded instrument that captures the multidimensional aspects of nurses’ nutritional knowledge, attitudes, and practices within advanced CKD management, thereby supporting educational planning and improving patient outcomes.

### Aim of the Study

This study aimed to develop and test the psychometric properties of the NECN-ESRD scale, designed to assess nephrology nurses’ competencies in nutritional care for patients with ESRD. Although primarily a methodological validation, the availability of a valid and reliable instrument is intended to support future clinical practice by informing educational planning, benchmarking professional competencies, and enhancing the consistency of nutritional care provided to patients with advanced CKD.

## 2. Materials and Methods

### 2.1. Design

This was a methodological study for the development and psychometric validation of the NECN-ESRD, structured into five sequential phases: construct definition and item generation, expert consultation and item revision, quantitative content validity analysis, pilot testing for comprehensibility, and final content validation prior to psychometric testing (validity and reliability analyses).

### 2.2. Setting and Sample

The study was conducted between August and September 2025 with registered nurses working in nephrology and dialysis clinical settings across Italy. The final sample achieved complete national geographic coverage, with representation from all 20 Italian regions. The distribution, however, was unbalanced, with the Northern regions accounting for the majority of participants (72.0%). Participants were recruited from nephrology wards, hemodialysis and peritoneal dialysis units, kidney transplant services, and nephrology palliative care programs. A non-probability purposive and snowball sampling strategy was adopted, leveraging the Italian Society of Nephrology Nurse (SIAN) network of affiliated professionals and relevant social media platforms, to ensure a national representation. The sampling strategy aimed to recruit nurses with diverse clinical backgrounds and levels of experience to ensure variability within the target population. Inclusion criteria encompassed all nurses directly involved in the care of patients with CKD, whereas those not working in nephrology contexts, those without direct clinical responsibilities in the care of CKD patients, or those engaged exclusively in administrative, managerial, or non-clinical roles were excluded.

### 2.3. Instrument Development

**Figure 1:**
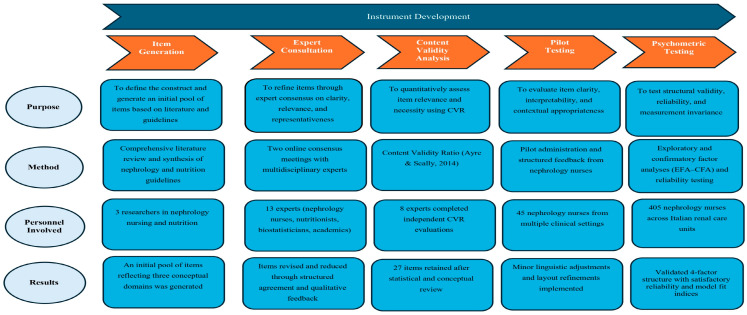
Sequential Phases of Development and Validation of the NECN-ESRD Questionnaire.

The development and validation of the NECN-ESRD questionnaire followed a rigorous multi-phase methodological process conducted in accordance with the COnsensus-based Standards for the selection of health Measurement INstruments (COSMIN) recommendations [29]. The overall development process was organized into five sequential phases, each aimed at progressively strengthening the conceptual, methodological, and psychometric foundations of the instrument (Graphical representation summary in Figure 1).

The NECN-ESRD questionnaire was originally developed in Italian for use among nurses working in nephrology and dialysis settings in Italy. The final validated version of the NECN-ESRD questionnaire is provided in Supplementary File S1.

#### 2.3.1. Phase I—Item Pooling

An extensive review of the scientific literature, international clinical guidelines in nephrology and nutrition, and existing assessment tools was conducted. The construct was defined as the level of nursing competencies, knowledge, and attitudes related to the nutritional management of patients with ESRD. In its initial version, the questionnaire comprised 27 items organized into three main sections of nine items each, designed to explore the following conceptual domains: (1) Perceived importance of nutritional interventions—the extent to which nurses believe specific nutritional care actions should be implemented in the management of patients with ESRD; (2) Perceived professional competencies—nurses’ self-assessment (or assessment of the nursing team) of their ability to perform these interventions in clinical practice; and (3) Frequency of nutritional care practices—how often these interventions are actually carried out in daily clinical settings [30,31].

#### 2.3.2. Phase II—Expert Panel

A multidisciplinary panel of 13 experts was invited to review the questionnaire draft. The panel was composed of three members of the SIAN research group, three members of the SIAN Nutrition Group, two biostatisticians with expertise in psychometric analysis, two clinical nutritionists specialized in CKD, two academic experts in nursing science, and one SIAN professional with expertise in palliative care. Of the 13 invited experts, eight participated in two structured online meetings (approximately 60 min each), during which each item was critically assessed for its relevance, linguistic clarity, representativeness, and domain coverage. The panel consisted of two nutritionists, one biostatistician, one palliative care nurse, two nephrology and dialysis nurses, two nurses from the SIAN research group, and one university faculty member, ensuring multidisciplinary expertise aligned with the instrument’s scope. Although participation was slightly lower than initially planned, this number remained within the methodologically acceptable range (5–10 experts) recommended for content validation [29,32,33]. Previous evidence indicates that involving at least six experts is sufficient to achieve a reliable consensus if agreement levels reach 70–80% [34], and COSMIN guidelines support the adequacy of involving 5–7 experts for assessing item relevance, clarity, and completeness [29]. A participation rate of approximately 70% of invited experts is also considered methodologically robust [34,35]. All decisions on item revision, removal, or addition were reached through structured consensus and documented in a decision log.

#### 2.3.3. Phase III—Content Validity

Quantitative analysis of content validity was conducted using the Content Validity Ratio (CVR), originally proposed by Lawshe [36] and refined by Ayre and Scally [37]. A separate expert panel included 13 nephrology and dialysis nurses, selected for their direct clinical expertise and familiarity with the construct, and who also served as members of the Research Board of SIAN. Each expert independently evaluated the items by classifying them as “essential,” “useful but not essential,” or “not necessary” for measuring the construct. Items with CVR values below the critical threshold (CVR ≥ 0.54) were revised or removed to ensure conceptual precision and alignment with the intended construct [37,38,39].

#### 2.3.4. Phase IV—Pilot Testing

The revised version of the questionnaire was administered to a convenience sample of 45 nurses working in nephrology setting. Participants were asked to complete the questionnaire and to provide structured feedback on the clarity, interpretability, and contextual appropriateness of the items. Based on this feedback, minor linguistic refinements were made to improve the clarity and comprehensibility of the instrument, in line with COSMIN recommendations [29].

#### 2.3.5. Phase V—Psychometric Testing

After pilot testing the final draft of the NECN-ESRD questionnaire was administered in a large-scale survey for final validation. Data were collected through a secure online platform, which allowed participants to complete the survey only once by preventing multiple submissions from the same IP address. Participation was voluntary and anonymous, and informed consent was obtained electronically prior to access.

### 2.4. Instrument

The self-report NECN-ESRD included 27 items designed to assess three complementary dimensions of nutritional care competence in nephrology nursing: (1) what nurses believe should be performed, (2) what they feel able to perform, and (3) what is actually performed in their clinical setting. Each item is rated on a 5-point Likert scale (1 = strongly disagree to 5 = strongly agree). The 27 items cover essential nutritional care interventions for patients with ESRD, including monitoring nutrient intake, identifying malnutrition, collaborating with multidisciplinary professionals, providing nutritional education, assessing electrolytes, evaluating hydration status, detecting taste and smell alterations, and managing nutrition-related complications. Domain scores were computed as the mean of the items within each of the three dimensions (“should be performed,” “able to perform,” and “performed in clinical practice”). The use of mean scores preserved the original 1–5 Likert response range and allowed comparability across domains with different numbers of items. In line with the multidimensional structure of the instrument, no overall total score was calculated. Participants’ sociodemographic and professional information was also collected, including age, gender, level of education, years of professional experience, type of healthcare facility, unit or department of employment, and duration of work in the current unit. These data were used to characterize the sample and to explore potential differences in questionnaire scores based on personal and professional variables.

### 2.5. Statistical Analysis

To cross-validate the latent structure, the total sample was randomly split into two independent subsamples, with 60% of cases allocated to an exploratory dataset and the remaining 40% to a confirmatory dataset [40].

Within the exploratory subsample, an EFA was performed using maximum likelihood extraction and oblimin rotation to identify the underlying factor structure. Data adequacy was checked with the Kaiser–Meyer–Olkin (KMO) measure and Bartlett’s test of sphericity. The number of factors to retain was guided by Monte Carlo simulated parallel analysis and theoretical interpretability [41,42].

The factor structure emerging from the EFA was then tested in the confirmatory subsample using CFA. Both first-order and second-order CFA models were specified and estimated with the Weighted Least Squares Mean and Variance-adjusted (WLSMV) method [43]. Model adequacy was evaluated using multiple fit indices: the Comparative Fit Index (CFI), Tucker–Lewis Index (TLI), Root Mean Square Error of Approximation (RMSEA) with 90% confidence interval (90%CI), and the Standardized Root Mean Square Residual (SRMR). Conventional thresholds were applied to judge model fit: CFI and TLI ≥ 0.90 for acceptable fit and ≥0.95 for excellent fit, RMSEA ≤ 0.08 for acceptable fit and ≤0.05 for excellent fit, and SRMR ≤ 0.08 as an indicator of adequate fit [44,45].

Internal consistency and reliability of the latent factors were assessed through Cronbach’s alpha, McDonald’s omega (total and hierarchical), explained common variance (ECV), composite reliability (CR), and signal-to-noise ratio (S/N) [46,47,48]. Test–retest reliability (stability) was evaluated in a subsample of 21 participants who completed the questionnaire twice, with an interval of approximately seven days between administrations. Stability was assessed using the Intraclass Correlation Coefficient (ICC). Specifically, we computed a two-way random effects model, single measures, absolute agreement definition [ICC (2,1)], as recommended by Koo & Li [49]. The 95% confidence intervals (95% CIs) were estimated based on the F distribution of mean squares from a two-way ANOVA model.

Finally, measurement invariance of the best-fitting CFA model was examined across educational level (basic vs. advanced) and professional experience (<10 vs. ≥10 years). Following the standard hierarchical sequence, configural, metric, scalar, and strict invariance models were tested. Invariance was evaluated by changes in fit indices, with ΔCFI < 0.01 and ΔRMSEA < 0.015 considered evidence of invariance across groups [50,51]. All analyses were conducted in R (version 4.5.0).

### 2.6. Sample Size Estimation

The minimum sample size required for the CFA was estimated using a Markov-Chain Monte Carlo simulation [52,53] in R (4.5.0) with the lavaan package. The NECN-ESRD instrument was theoretically conceptualized to assess three core domains of nutritional care competencies in nephrology nursing. Based on this expected factorial structure, a population model with three correlated latent factors (nine indicators per factor, standardized loadings = 0.60, inter-factor correlations = 0.30) was specified to guide the Monte Carlo simulation.

Simulated datasets were generated for sample sizes ranging from 150 to 300 (in steps of 10, *n* = 100 replications). Across the simulated conditions, model fit indices improved steadily with increasing sample size. At smaller samples (*n* ≈ 150–170), the mean RMSEA ranged from 0.020 to 0.021 and the mean CFI/TLI were around 0.97. Fit indices reached optimal and stable values from *n* ≈ 180–200 onward, with mean CFI ≈ 0.98–0.99, TLI ≈ 0.98–0.99, RMSEA ≈ 0.01–0.02, and SRMR ≈ 0.06–0.08. Beyond *n* = 220, additional gains were minimal, indicating convergence of the model’s performance. Based on these results, an optimal target of 220–240 participants was defined for the CFA, with a prudential minimum of 180 to ensure adequate model stability and reliable parameter estimation. Considering a 60–40% split between exploratory and confirmatory subsamples, the total recommended sample size was approximately 450 participants (EFA ≈ 270–300; CFA ≈ 180–200). This target also provides sufficient power for potential measurement invariance analyses across groups (≈150 cases per subgroup).

### 2.7. Ethical Considerations

The study was conducted in accordance with the principles of the Declaration of Helsinki and national regulations governing research involving human participants. Participation was voluntary and anonymous, and all participants provided electronic informed consent prior to completing the questionnaire. Because data collection was conducted via an anonymous online survey and no patient data or clinical interventions were involved, no additional institutional permissions were required. The study protocol was reviewed and approved by the Scientific Committee of the SIAN under approval number SIAN.RIC 01/2025, dated 30 April 2025. All collected data were accessible only to the research team and were stored in a secure, password-protected database. All participants gave their consent to the processing of their personal data in an anonymous and computerized format.

## 3. Results

### 3.1. Characteristics of Participants

The sample included 405 nurses. Most participants were female (*n* = 337, 83.2%), employed in nephrology and dialysis units located in northern Italy (72.0%), with lower representation from the central (15.5%) and southern regions or islands (12.5%). Detailed descriptive sample statistics are reported in Table 1. For psychometric analyses, the total sample (N = 405) was randomly split into two independent subsamples: one used for the EFA (*n* = 243) and one for CFA (*n* = 162). The two subsamples showed comparable socio-demographic characteristics (gender distribution, age, geographical area, education, and years of experience), supporting the methodological robustness of the split-sample approach and ensuring comparability between EFA and CFA results.

### 3.2. Content Validity

Table 2 reports the CVR for each item of the NECN-ESRD questionnaire, based on expert panel evaluation. The CVRs ranged 0.54–1.00. The critical CVR value for 13 experts is 0.54. Items with CVR ≥ 0.54 were considered acceptable [33,34].

### 3.3. Exploratory Factor Analysis

Data were suitable for EFA: χ^2(351)^ = 8699.42, *p* < 0.001, KMO = 0.91 (ranging 0.88–0.94) LRT remained significant, χ^2(249)^ = 885.82, *p* < 0.001, with RMSEA = 0.103, 90% CI [0.096, 0.110], and TLI = 0.822. Residual-based indices suggested adequate reproduction of the observed correlations (RMSR = 0.04; corrected RMSR = 0.05.

Factor 1 (F1) labelled as “Recommendations” represents the normative dimension of nutritional care, namely what nurses believe should be implemented within their own nephrology clinical context to ensure appropriate nutritional management in accordance with professional standards. Factor 2 (F2) labelled as “Attitudes” captures nurses’ perceived ability and self-efficacy to perform nutritional interventions within their clinical context. Factor 3 (F3) labelled as “Practice” reflects the extent to which nutritional care activities are actually implemented in daily clinical settings. Factor 4 (F4) labelled as “Advanced Competencies” encompasses higher-level clinical skills, such as the assessment of sensory alterations, the management of nutritional complications, and the application of evidence-based guidelines. The loadings for the four-factor solution are reported in Table 3. Clinically, this structure indicates that nutritional competence in nephrology nursing is multidimensional, encompassing normative knowledge, perceived self-efficacy, actual practice, and advanced clinical skills.

### 3.4. Confirmatory Factor Analysis

**Figure 2:**
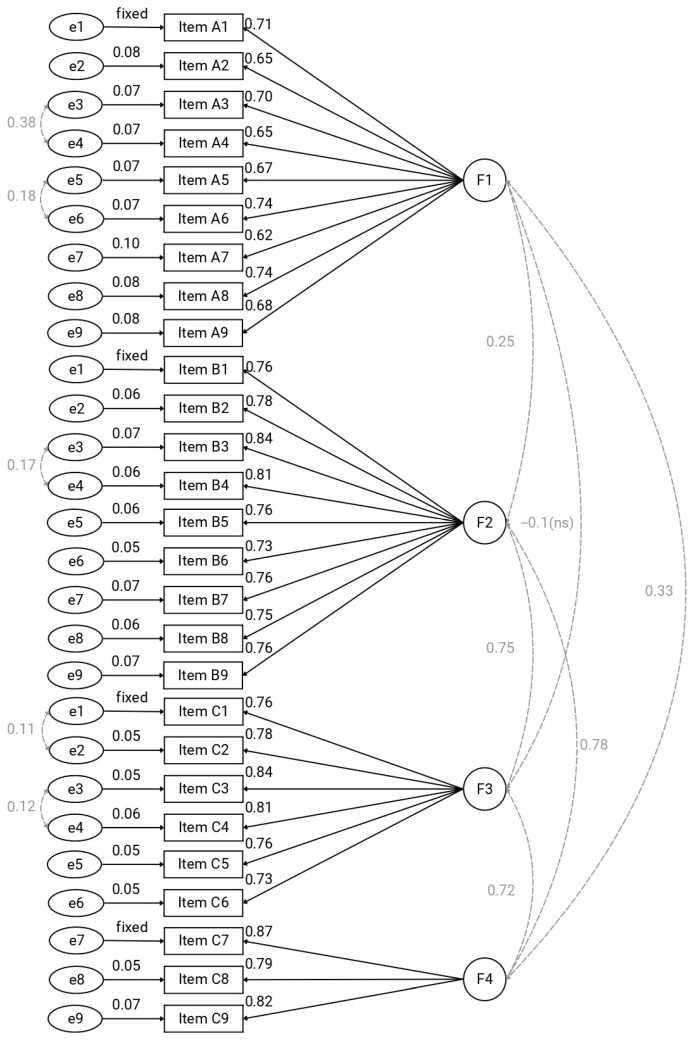
First-order four-factor CFA model of the NECN-ESRD scale. Note: F1: Recommendations; F2: Attitudes; F3: Practice, F4: Advanced Competencies. Factor loadings for each item are standardized. The first loading of each factor was fixed at λ = 1.00 for identification purposes.

First, a first-order four–factor model was estimated (Figure 2). Model fit was satisfactory, χ^2^_(313)_ = 560.40, *p* < 0.001, CFI = 0.995, TLI = 0.994, SRMR = 0.08, RMSEA = 0.07, 90% CI [0.061, 0.079]. First-order model explained 59.3% of variance. Overall, these results support the robustness of the four-dimensional structure and its coherence as a measurement model in nephrology nursing practice.

**Figure 3:**
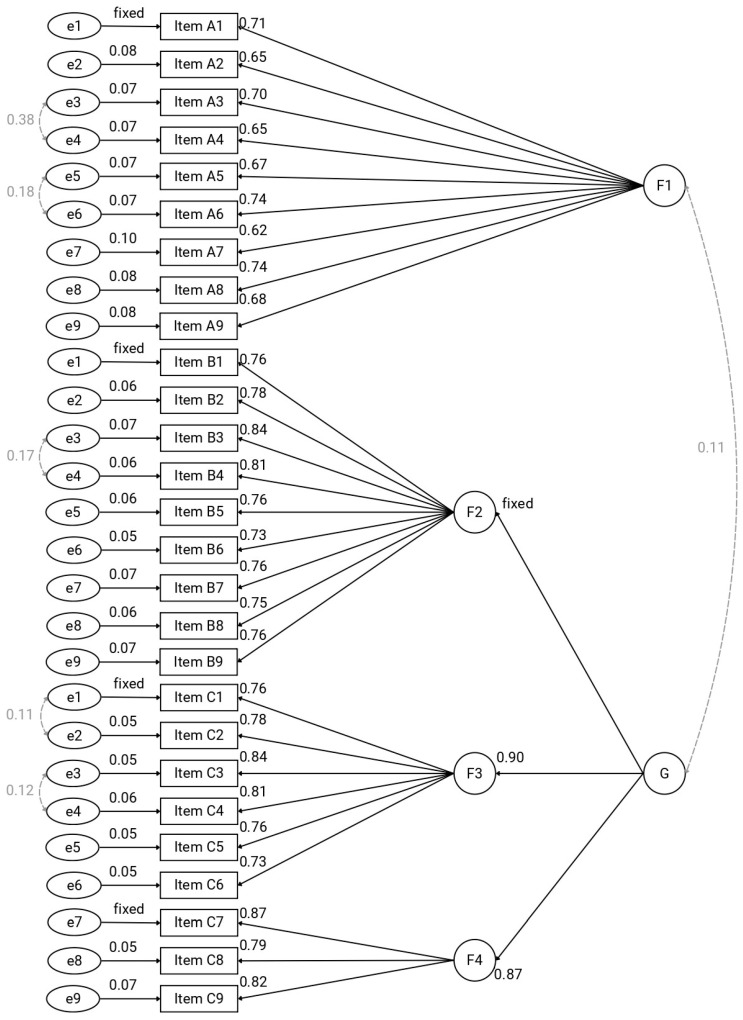
Second-order CFA model with a general factor (G) underlying factors F2–F4. Note: G: General factor; F1: Recommendations; F2: Attitudes; F3: Practice; F4: Advanced Competencies. Standardized factor loadings are reported. The first loading of each factor was fixed at λ = 1.00 for identification purposes.

Next, a second-order model (Figure 3) was specified in which a higher–order factor (G) accounted for the covariances among three of the first–order factors (F2, F3, and F4), while F1 was allowed to correlate with the higher–order factor (r = 0.108). This second-order solution demonstrated an excellent fit to the data, χ^2^_(320)_ = 362.00, *p* = 0.053, CFI = 0.994, TLI = 0.993, SRMR = 0.079, and RMSEA = 0.029, 90% CI [0.000, 0.042]. Second-order model explained 68.2% of variance.

### 3.5. Reliability and Test–Retest Stability

All reliability indices showed excellent performance across both first- and second-order latent factors of the NECN-ESRD. Internal consistency was high, with Cronbach’s α values ranging from 0.86 to 0.94 and McDonald’s omega total ranging from 0.87 to 0.96, indicating strong homogeneity of items within each dimension (Table 4 and Table 5). Composite Reliability coefficients were likewise robust (CR = 0.87–0.94). ECV values confirmed well-defined latent factors for F1, F2, and F3, while the hierarchical structure was further supported by the high reliability of the second-order factor (ωt = 0.96; CR = 0.93).

Analysis of test–retest reliability also demonstrated excellent temporal stability: the intraclass correlation coefficient indicated perfect agreement (ICC [2, 1] = 1.00; 95% CI [1.00–1.00]). Taken together, reliability results indicate that NECN-ESRD scores are highly consistent and stable over a short retest interval, supporting their use for competency profiling and educational evaluation.

### 3.6. Measurement Invariances

As shown in Table 6, the measurement invariance analysis indicated that the NECN-ESRD maintained a stable factorial structure across educational levels and years of experience. Further constraints on factor loadings, intercepts, and residuals produced only negligible variations in fit indices, supporting both metric and scalar invariance across groups.

After inspecting the modification indices, the model was re-estimated by allowing the residuals of five pairs of items to covary namely ItemB3 with ItemB5, C3 with C4, B6 with B7, A5 with A6, and A3 with A4. With these correlations included, the configural model yielded an acceptable though not fully optimal fit. Imposing equality constraints on the factor loadings (metric invariance) did not affect the fit appreciably, and adding constraints on the intercepts (scalar invariance) produced only a trivial decrease. Finally, when residual variances were also constrained (strict invariance) the model remained stable. Across these successive steps the change in CFI never exceeded the recommended cut-off of 0.01, indicating that metric, scalar and even strict invariance can be assumed across the two experience groups. Although the absolute indices suggest a fit slightly below conventional cut-offs (for example CFI just under 0.90 and RMSEA slightly above 0.08), the stability of the fit measures across the increasingly constrained models supports the conclusion that the measurement structure operates equivalently in both groups, permitting meaningful comparison of latent means. This invariance suggests that the scale measures the same constructs across education and experience groups, enabling fair comparisons between different nursing profiles.

## 4. Discussion

This study aimed to develop and validate the NECN-ESRD questionnaire, a context-specific tool designed to assess nurses’ perceived competencies, training, and educational needs in the nutritional management of ESRD patients. Following COSMIN recommendations [29], the instrument was created through a rigorous multiphase process that included construct definition, item generation, expert review, quantitative content validation, pilot testing, and psychometric evaluation using EFA and CFA. During content validation, a multidisciplinary panel evaluated item clarity, relevance, and representativeness. High CVR (0.54–1.00) reflected strong expert consensus, confirming conceptual accuracy and clinical applicability [38,39,54,55]. This foundation is essential because nutritional care in ESRD requires both technical and relational competencies, and nurses often serve as the first professionals to detect changes in patients’ nutritional status [54,56,57]. The emergence of four dimensions: Recommendations, Attitudes, Practice and Advanced Competencies, highlights that nutritional care in nephrology is not only technical but also motivational, relational and organizational. These domains reflect the multidimensional nature of the nutritional process in dialysis care, where early risk identification, patient counselling, adherence support and interdisciplinary coordination are integral to safe and effective practice [54,56,57,58]. However, Recommendations (F1) showed non-significant correlations with the other first-order latent dimensions. This finding may indicate that Recommendations captures a distinct, normative aspect of competence rather than an integrated behavioral or attitudinal component. Conceptually, this domain represents what nurses believe “should” be done in nutritional care, an orientation toward professional standards and institutional protocols, rather than what they feel able to do (Attitudes), actually do (Practice), or perform at an advanced level (*Advanced Competencies*). This interpretive distinction suggests that the prescriptive or guideline-driven nature of Recommendations operates relatively independently from self-efficacy and behavioral engagement, a pattern also observed in other psychometric studies of professional competency frameworks [59,60]. Clinically, this gap between knowing and doing suggests the presence of structural and organizational barriers, such as limited time, workload, and fragmentation of responsibilities, that impede the translation of nutritional guidelines into everyday practice [61,62]. Strengthening organizational support, workflow integration and interdisciplinary collaboration may therefore be necessary to enable nurses to operationalize nutritional recommendations effectively [62]. These interpretations should be considered within the organizational and contextual characteristics of the sample. Differences in institutional models, regional healthcare organization, and workload distribution may influence how nutritional competencies are perceived and enacted in practice. As such, the identified patterns are most representative of settings with similar structural features.

The high internal consistency found supports its usefulness as a tool for competency assessment, educational planning and professional development [63,64,65]. Unlike more general international instruments assessing nursing competence or nutritional knowledge, the NECN-ESRD was specifically designed for the nephrology context and conceptualizes nutritional care as a multidimensional construct. Future cross-cultural validation studies may further clarify its positioning within the broader international landscape. However, because the retest interval was short, caution is warranted when considering the tool for long-term monitoring of competence [49]. Using separate subscale scores allows educators and managers to identify whether gaps arise from insufficient knowledge, motivational issues or organizational constraints, enabling targeted interventions at both the individual and system level [66]. This invariance supports the instrument’s use in heterogeneous nephrology teams, allowing fair comparisons of competence across units and professional profiles [67,68]. In this sense, the NECN-ESRD can serve as a practical decision-making tool for nurse managers, supporting workforce planning and helping to identify areas where additional training, supervision, or organizational adjustments may be required [25]. Moreover, integrating the scale into routine evaluation pathways could facilitate continuous monitoring of nutritional care processes and promote a more systematic approach to improving patient outcomes in dialysis settings [58,69].

From a clinical perspective, the NECN-ESRD can contribute to earlier detection of nutritional decline by highlighting gaps in nurses’ assessment skills and confidence, which are essential for identifying subtle changes in appetite, body composition, or functional status in ESRD patients [69]. Strengthening these competencies may help reduce the prevalence of protein-energy wasting and support more timely dietetic referrals, ultimately improving the stability and safety of dialysis treatments [57]. Within the present study design, these potential clinical benefits remain theoretical, as no objective patient outcome measures were collected.

At the organizational level, the tool may support standardization of nutritional practices within dialysis units, ensuring that evidence-based interventions are consistently implemented regardless of staffing patterns or workload variability [5]. Its use within clinical governance frameworks could facilitate auditing, benchmarking across centers, and the development of shared protocols that enhance coordination between nurses, dietitians, and nephrologists [58].

From an educational standpoint, the NECN-ESRD offers a structured framework that can guide the design of targeted training programs, ECM activities, and competency-based curricula in nephrology nursing [70]. By identifying specific learning needs in areas such as patient counselling, advanced assessment, or clinical reasoning, the instrument can support personalized educational pathways and promote the progressive acquisition of specialist nutritional competencies required in dialysis care [66].

### 4.1. Implication for Practice and Assistance

The NECN-ESRD provides several practical implications for nephrology nursing. First, the scale can support the early identification of competence gaps in nutritional assessment, counselling and advanced monitoring, guiding targeted clinical mentoring and structured training pathways for nurses working in dialysis care [71]. Second, by mapping the distribution of competencies across units, the tool can assist nurse leaders in optimizing workforce planning, aligning skill mix with patient needs, and supporting the development of advanced practice roles in nutrition management [72]. Third, incorporating the NECN-ESRD into routine quality cycles could provide nephrology teams with a structured system to monitor adherence to recommended nutritional practices over time, promptly identify deviations from standards, and coordinate shared corrective actions among nurses, dietitians, and nephrologists. This approach may enhance the clarity of communication and strengthen interdisciplinary integration, particularly in situations that require complex relational competencies [65]. Overall, the scale offers a structured framework that can enhance clinical decision-making, support organizational development, and ultimately contribute to safer and more effective nutritional care for ESRD patients.

### 4.2. Strengths and Limitations

This study presents several strengths. The NECN-ESRD was developed following internationally recognized psychometric guidelines (COSMIN framework) and supported by a rigorous multi-phase design including expert review, pilot testing, and large-scale validation. Nonetheless, some limitations should be acknowledged. The use of non-probabilistic sampling may have introduced selection bias, and the predominance of participants from Northern Italy (72%) may limit geographic representativeness. In addition, the short data collection period (August–September 2025) may have reduced seasonal or institutional variability, and potential confounding factors related to healthcare settings were not explicitly examined. First, the psychometric validation was based on a single-timepoint design, which does not allow assessment of temporal stability or responsiveness to educational or organizational interventions. Moreover, the short test–retest interval (approximately one week) supports interpretation of stability estimates as short-term rather than long-term. Longitudinal studies are needed to determine whether the NECN-ESRD can detect changes in competence over time. Second, the exclusive reliance on self-reported measures introduces risks of social desirability and recall bias and does not confirm whether perceived competence aligns with actual clinical performance. Additionally, because the study relied solely on self-reported data, future validation should incorporate complementary sources such as observational assessments or clinical performance measures. Third, although internal consistency was strong, the Recommendations dimension showed weak correlations with the higher-order factor, suggesting partial conceptual independence. Although the Recommendations domain behaved consistently with its conceptual definition, future studies may explore whether targeted item refinements could enhance its alignment with the broader competency framework. Fourth, the sample was nationally homogeneous, which may limit generalizability to contexts with different organizational structures, educational backgrounds, or dialysis models. Multicenter and international validation studies are recommended to verify factorial stability across diverse healthcare systems. Fifth, full scalar invariance across subgroups was not achieved, indicating potential differences in item interpretation among nurses with varying levels of experience. This may limit direct comparisons of mean scores across groups and highlights the need for replication in larger and more heterogeneous samples. Finally, the study did not include objective indicators of nutritional care quality; therefore, future research should examine how perceived competence relates to measurable clinical processes and outcomes. Future research should integrate multimethod approaches combining self-report instruments with observational and clinical outcome data to enhance the predictive and practical utility of the NECN-ESRD.

## 5. Conclusions

The NECN-ESRD questionnaire was developed through a rigorous and methodologically sound process and provides a valid tool to assess nurses’ competencies in nutritional care for patients with CKD and ESRD. The scale captures essential dimensions of clinical practice—recommendations, attitudes, practice, and advanced competencies—highlighting areas in which nurses may require support or targeted training. Future research should examine its responsiveness to educational interventions and its applicability across diverse nephrology settings. Future research should include cross-cultural validation studies, explore the applicability of the NECN-ESRD in other clinical contexts beyond nephrology, and examine its relationship with objective clinical indicators and patient-related outcomes.

## Figures and Tables

**Table 1:** Characteristics of study participants (*n* = 405 nurses).

Variables	Values
Gender	N (%)
Female	337 (83.2)
Male	68 (16.8)
Region	N (%)
North	288 (72.0)
Centre	62 (15.5)
South/Islands	50 (12.5)
Education	N (%)
Bachelor’s Degree	256 (63.2)
Postgraduate	149 (36.8)
Age (years) M (SD), range	47.20 (9.80), 22–66
Experience (years) M (SD), range	22.6 (7.35), 0–41
Clinical setting	N (%)
Hemodialysis	327 (80.7)
Peritoneal dialysis	18 (4.4)
Nephrology ward	31 (7.7)
Transplant area	5 (1.2)
Other	24 (5.9)

Note: Values are expressed as N (%) unless otherwise indicated. Legend. M = mean; SD = standard deviation.

**Table 2:** CVR of the NECN-ESRD Questionnaire Items.

Item	CVR
Item1	0.54
Item2	1.00
Item3	0.54
Item4	0.85
Item5	0.69
Item6	0.85
Item7	0.54
Item8	0.54
Item9	0.85

Note: CVR = Content Validity Ratio.

**Table 3:** Standardized factor loadings of the four-factor exploratory factor analysis.

Item	F1	F2	F3	F4	h^2^	u^2^	Complexity
ItemA1	**0.76**	0.00	0.05	−0.16	0.52	0.48	1.1
ItemA2	**0.78**	0.02	0.00	−0.02	0.61	0.39	1.0
ItemA3	**0.73**	0.15	−0.11	0.00	0.59	0.41	1.1
ItemA4	**0.80**	0.05	−0.02	−0.11	0.61	0.39	1.0
ItemA5	**0.84**	−0.04	0.02	0.02	0.71	0.29	1.0
ItemA6	**0.84**	−0.08	−0.04	0.08	0.74	0.26	1.0
ItemA7	**0.56**	−0.02	0.17	−0.10	0.30	0.70	1.3
ItemA8	**0.80**	0.00	−0.07	0.14	0.72	0.28	1.1
ItemA9	**0.77**	−0.07	0.06	0.05	0.61	0.39	1.0
ItemB1	0.04	**0.62**	0.26	−0.04	0.58	0.42	1.4
ItemB2	0.03	**0.80**	0.21	−0.15	0.74	0.26	1.2
ItemB3	−0.03	**0.81**	0.01	0.04	0.69	0.31	1.0
ItemB4	−0.03	**0.81**	−0.05	0.09	0.68	0.32	1.0
ItemB5	0.07	**0.46**	−0.12	0.54	0.66	0.34	2.1
ItemB6	0.09	**0.42**	−0.22	0.62	0.68	0.32	2.1
ItemB7	0.08	**0.56**	0.32	−0.04	0.56	0.44	1.7
ItemB8	0.15	**0.40**	−0.07	0.47	0.57	0.43	2.2
ItemB9	0.02	**0.68**	0.22	0.08	0.70	0.30	1.2
ItemC1	0.03	0.08	**0.75**	0.04	0.65	0.35	1.0
ItemC2	−0.03	0.20	**0.76**	0.03	0.77	0.23	1.1
ItemC3	−0.02	0.16	**0.61**	0.16	0.61	0.39	1.3
ItemC4	0.01	0.08	**0.53**	0.36	0.63	0.37	1.8
ItemC5	0.06	−0.05	0.30	0.72	**0.78**	0.22	1.4
ItemC6	0.04	−0.07	0.17	**0.80**	**0.75**	0.25	1.1
ItemC7	0.01	0.03	0.83	0.02	**0.73**	0.27	1.0
ItemC8	0.07	−0.05	0.31	0.69	**0.74**	0.26	1.4
ItemC9	−0.04	0.10	**0.69**	0.18	**0.69**	0.31	1.2

Note: F1—Recommendations: items A1–A9; F2—Attitudes: items B1–B9; F3—Practice: items C1–C6; F4—Advanced Competencies: items C7–C9; h^2^: Communality; u^2^: Uniqueness; Complexity: item cross-loading index. Loadings bold are considered salient.

**Table 4:** Internal consistency and reliability indices for the four latent factors.

Factor	Cronbach’s α	Omega Total (ωt)	Omega Hierarchical (ωh)	ECV	CR	S/N
F1	0.92	0.94	0.83	0.76	0.89	11
F2	0.92	0.95	0.81	0.71	0.93	12
F3	0.93	0.96	0.86	0.79	0.94	13
F4	0.89	0.89	0.03	0.03	0.87	8.3

Note: α = Cronbach’s alpha; ωt = McDonald’s omega total; ωh = omega hierarchical (from psych:omega, not applicable for the second-order factor); CR = composite reliability; ECV = Explained Common Variance; S/N = Signal-to-noise ratio, indicating the relative strength of the common latent factor compared with residual variance; higher values reflect a stronger latent signal. Reliability indices refer to first-order factors considered as stand-alone dimensions.

**Table 5:** Internal consistency and reliability indices for first- and second-order factors.

Factor	Cronbach’s α	Omega Total (ωt)	Omega Hierarchical (ωh)	CR	AVE
F1	0.89	0.88	0.83	0.89	0.46
F2	0.93	0.93	0.81	0.93	0.60
F3	0.94	0.94	0.86	0.94	0.72
F4	0.86	0.87	0.03	0.87	0.69
G	0.93	0.96	–	0.93	0.63

**Note**: α = Cronbach’s alpha; ωt = McDonald’s omega total; ωh = omega hierarchical (from psych::omega, not applicable for the second-order factor); CR = composite reliability (from semTools::reliability); AVE = average variance extracted. Reliability indices for F1–F4 differ from those reported in Table 4 because they are derived from the final hierarchical model including a second-order general factor.

**Table 6:** Measurement Invariances.

Invariances	CFI	RMSEA	SRMR	ΔCFI
Ed. Level (Basic vs. Advanced)				
Configural	0.991	0.033	0.084	–
Metric (loadings)	0.988	0.036	0.086	−0.002
Scalar (loadings + intercepts)	0.988	0.036	0.087	0.000
Strict (loadings + intercepts + residuals)	0.988	0.035	0.089	0.000
Experience (<10 vs. >10 years)				
Configural	0.867	0.095	0.082	–
Metric (loadings)	0.867	0.093	0.085	0.000
Scalar (loadings + intercepts)	0.866	0.092	0.085	−0.001
Strict (loadings + intercepts + residuals)	0.858	0.093	0.087	−0.008

Note: CFI = Comparative Fit Index; RMSEA = Root Mean Square Error of Approximation; SRMR = Standardized Root Mean Square Residual; ΔCFI = Change in Comparative Fit Index between successive models.

## Data Availability

The data analyzed in this study were provided in aggregated form by corporate sources and cannot be publicly shared due to ethical, contractual, and legal constraints. Specific data access requests may be considered on a case-by-case basis, subject to authorization by the relevant companies. The corresponding authors may act as intermediaries to facilitate contact with the data owners and assess the feasibility of such requests, in full compliance with applicable regulations.

## Supplementary Materials

The following supporting information can be downloaded at: https://www.mdpi.com/article/10.3390/nursrep16030078/s1, Supplementary File S1. Final version of the NECN-ESRD questionnaire (Italian).

## Public Involvement Statement

No public involvement in any aspect of this research.

## Guidelines and Standards Statement

This manuscript was drafted in accordance with the COSMIN (COnsensus-based Standards for the Selection of Health Measurement INstruments) reporting guideline for studies on measurement properties of health-related instruments [27].

## Abbreviations

The following abbreviations are used in this manuscript:

SIAN	Italian Society of Nephrology Nurse
NECN-ESRD	Nursing Education and Competencies in Nutrition for Patients with CKD in ESRD
CKD	Chronic Kidney Disease
ESRD	End Stage Renal Disease
CVR	Content Validity Ratio
EFA	Exploratory Factor Analysis
CFA	Confirmatory Factor Analysis
BIA	Bioelectrical Impedance Analysis
IDWG	Interdialytic Weight Gain
QoL	Quality of Life
KDOQI	Kidney Disease Outcomes Quality Initiative
KMO	Kaiser–Meyer–Olkin Measure of Sampling Adequacy
WLSMV	Weighted Least Squares Mean and Variance Adjusted
CFI	Comparative Fit Index
TLI	Tucker–Lewis Index
RMSEA	Root Mean Square Error of Approximation
SRMR	Standardized Root Mean Square Residual
ECV	Explained Common Variance
ICC	Intraclass Correlation Coefficient

## Appendix A

Italian Society of Nephrology Nurse (SIAN) Research Group (contributed as authors of the manuscript):

1. **Domenica Gazineo:** Italian Society of Nephrology Nurse (SIAN) Research Group, Via Capotesta 1/30, 07026 Olbia, Italy; Governo Clinico e Qualità, IRCCS Azienda Ospedaliero-Universitaria di Bologna, Bologna, Italy; domenica.gazineo3@unibo.it;
2. **Lea Godino:** Italian Society of Nephrology Nurse (SIAN) Research Group, Via Capotesta 1/30, 07026 Olbia, Italy; Medical Genetics Unit, IRCCS Azienda Ospedaliero-Universitaria di Bologna, Bologna, lea.godino2@unibo.it;
3. **Immacolata De Simone:** Italian Society of Nephrology Nurse (SIAN) Research Group, Via Capotesta 1/30, 07026 Olbia, Italy; desimone.titti@gmail.com;
4. **Giada Vrenna:** Italian Society of Nephrology Nurse (SIAN) Research Group, Via Capotesta 1/30, 07026 Olbia, Italy; giadavrenna@hotmail.com;
5. **Eleonora Faraglia:** Italian Society of Nephrology Nurse (SIAN) Research Group, Via Capotesta 1/30, 07026 Olbia, Italy; ele80ip@gmail.com;
6. **Alessandro Pizzo:** Italian Society of Nephrology Nurse (SIAN) Research Group, Via Capotesta 1/30, 07026 Olbia, Italy; alessandro.pizzo@fmc-ag.com;
7. **Silvia Cappelletti:** Italian Society of Nephrology Nurse (SIAN) Research Group, Via Capotesta 1/30, 07026 Olbia, Italy; silviacappelletti74@outlook.com.

## References

[1] Deng L., Guo S., Liu Y., Zhou Y., Liu Y., Zheng X., Yu X., Shuai P. (2025). Global, regional, and national burden of chronic kidney disease and its underlying etiologies from 1990 to 2021: A systematic analysis for the Global Burden of Disease Study 2021. BMC Public Health.

[2] Marino C., Ferraro P.M., Bargagli M., Cascini S., Agabiti N., Gambaro G., Davoli M. (2020). Prevalence of chronic kidney disease in the Lazio region, Italy: A classification algorithm based on health information systems. BMC Nephrol..

[3] Bellasi A., Di Iorio B., Di Lullo L. (2020). Tackling chronic kidney diseases in the third millennium in Italy: How can digital health help the National Health System?. AboutOpen.

[4] De Nicola L., Donfrancesco C., Minutolo R., Lo Noce C., Palmieri L., De Curtis A., Iacoviello L., Zoccali C., Gesualdo L., Conte G. (2015). Prevalence and cardiovascular risk profile of chronic kidney disease in Italy: Results of the 2008–12 National Health Examination Survey. Nephrol. Dial. Transplant..

[5] Ikizler T.A., Burrowes J.D., Byham-Gray L.D., Campbell K.L., Carrero J.-J., Chan W., Fouque D., Friedman A.N., Ghaddar S., Goldstein-Fuchs D.J. (2020). KDOQI Clinical Practice Guideline for Nutrition in CKD: 2020 Update. Am. J. Kidney Dis..

[6] Ikizler T.A., Cuppari L. (2021). The 2020 Updated KDOQI Clinical Practice Guidelines for Nutrition in Chronic Kidney Disease. Blood Purif..

[7] Fouque D., Kalantar-Zadeh K., Kopple J., Cano N., Chauveau P., Cuppari L., Franch H., Guarnieri G., Ikizler T.A., Kaysen G. (2008). A Proposed Nomenclature and Diagnostic Criteria for Protein-Energy Wasting in Acute and Chronic Kidney Disease. Kidney Int..

[8] Koppe L., Fouque D., Kalantar-Zadeh K. (2019). Kidney Cachexia or Protein-Energy Wasting in CKD: Facts and Numbers. J. Cachexia Sarcopenia Muscle.

[9] Rashid I., Bashir A., Tiwari P., D’Cruz S., Jaswal S. (2021). Estimates of Malnutrition Associated with Chronic Kidney Disease Patients Globally and Its Contrast with India: An Evidence-Based Systematic Review and Meta-Analysis. Clin. Epidemiol. Glob. Health.

[10] Xi W.Z., Wu C., Liang Y.L., Wang L.L., Cao Y.H. (2023). Analysis of Malnutrition Factors for Inpatients with Chronic Kidney Disease. Front. Nutr..

[11] Starace E., De Pasquale G., Morenghi E., Crippa C., Matteucci S., Pieri G., Soekeland F., Gibbi S.M., Lo Cricchio G., Reggiani F. (2023). Hospital Malnutrition in the Medicine and Neurology Departments: A Complex Challenge. Nutrients.

[12] Sguanci M., Ferrara G., Morales Palomares S., Parozzi M., Godino L., Gazineo D., Anastasi G., Mancin S. (2024). Dysgeusia and Chronic Kidney Disease: A Scoping Review. J. Ren. Nutr..

[13] Morales Palomares S., Parozzi M., Ferrara G., Andreoli D., Godino L., Gazineo D., Anastasi G., Sguanci M., Mancin S. (2025). Olfactory Dysfunctions and Chronic Kidney Disease: A Scoping Review. J. Ren. Nutr..

[14] Ouirdani M., Boutib A., Azizi A., Chergaoui S., Saad E.M., Hilali A., Marfak A., Youlyouz-Marfak I. (2024). Impact of Nutrition Education on Various Health-Related Components of Hemodialysis Patients: A Systematic Review. Healthcare.

[15] Park G., Choi S. (2023). The Effects of a Tailored Dietary Education Program for Older Adult Patients on Hemodialysis: A Preliminary Study. Healthcare.

[16] Pernas A., Pires S., Gomes I., Fonseca C., Ramos A. (2025). Technological Nursing Interventions on Nutritional Status of Middle-Aged and Older Adults Undergoing Hemodialysis: A Systematic Review. Int. J. Nurs. Sci..

[17] Cupisti A., Ferretti V., D’Alessandro C., Petrone I., Di Giorgio A., Meola M., Panichi V., Conti P., Lippi A., Caprioli R. (2012). Nutritional Knowledge in Hemodialysis Patients and Nurses: Focus on Phosphorus. J. Ren. Nutr..

[18] Fletcher A., Carey E. (2011). Knowledge, Attitudes and Practices in the Provision of Nutritional Care. Br. J. Nurs..

[19] Ang W.L.S., Zhang D., Cai H., Chew H.S.J. (2025). Nurses’ Knowledge, Attitude and Practice in Nutrition Management of Hospitalised Adults: A Mixed-Methods Study. J. Clin. Nurs..

[20] Bonetti L., Bagnasco A., Aleo G., Sasso L. (2013). Validation of the Staff Attitudes to Nutritional Nursing Care Geriatric Scale in Italian. Int. Nurs. Rev..

[21] Barril G., Nogueira A., Alvarez-García G., Núñez A., Sánchez-González C., Ruperto M. (2022). Nutritional Predictors of Mortality after 10 Years of Follow-Up in Patients with Chronic Kidney Disease at a Multidisciplinary Unit of Advanced Chronic Kidney Disease. Nutrients.

[22] Swift L.M., Kearney L.N., Hyun A., Levett-Jones T.L., Bogossian F.E. (2025). Defining Registered Nurse Competence: A Contemporary Concept Analysis. Collegian.

[23] International Council of Nurses (ICN) (2020). Guidelines on Advanced Practice Nursing.

[24] American Association of Colleges of Nursing (AACN) (2021). The Essentials: Core Competencies for Professional Nursing Education.

[25] Wit R.F., de Veer A.J.E., Batenburg R.S., Francke A.L. (2023). International Comparison of Professional Competency Frameworks for Nurses: A Document Analysis. BMC Nurs..

[26] Shi Y.-X., Fan X.-Y., Han H.-J., Wu Q.-X., Di H.-J., Hou Y.-H., Zhao Y. (2013). Effectiveness of a Nurse-Led Intensive Educational Programme on Chronic Kidney Failure Patients with Hyperphosphataemia: Randomised Controlled Trial. J. Clin. Nurs..

[27] Arooj H., Aman M., Hashmi M.U., Nasir Z., Zahid M., Abbas J., Amjad N., Maryam S., Farhan K. (2025). The Impact of Nurse-Led Care in Chronic Kidney Disease Management: A Systematic Review and Meta-Analysis. BMC Nurs..

[28] Wei R., Lv H., Jiang G., Wang X., Zhang N., Guo S. (2024). Constructing a Competency Evaluation Index System for Nursing Positions in a Chronic Kidney Disease Management Centre. J. Multidiscip. Healthc..

[29] Mokkink L.B., de Vet H.C.W., Prinsen C.A.C., Patrick D.L., Alonso J., Bouter L.M., Terwee C.B. (2018). COSMIN Risk of Bias Checklist for Systematic Reviews of Patient-Reported Outcome Measures. Qual. Life Res..

[30] Mokkink L.B., Elsman E.B.M., Terwee C.B. (2024). COSMIN Guideline for Systematic Reviews of Patient-Reported Outcome Measures Version 2.0. Qual. Life Res..

[31] Terwee C.B., Prinsen C.A.C., Chiarotto A., Westerman M.J., Patrick D.L., Alonso J., Bouter L.M., de Vet H.C.W., Mokkink L.B. (2018). COSMIN Methodology for Evaluating the Content Validity of Patient-Reported Outcome Measures: A Delphi Study. Qual. Life Res..

[32] Krueger R.A., Casey M.A. (2014). Focus Groups: A Practical Guide for Applied Research.

[33] Lynn M.R. (1986). Determination and Quantification of Content Validity. Nurs. Res..

[34] Hasson F., Keeney S., McKenna H. (2000). Research Guidelines for the Delphi Survey Technique. J. Adv. Nurs..

[35] Keeney S., Hasson F., McKenna H. (2011). The Delphi Technique in Nursing and Health Research.

[36] Lawshe C.H. (1975). A Quantitative Approach to Content Validity. Pers. Psychol..

[37] Ayre C., Scally A.J. (2014). Critical Values for Lawshe’s Content Validity Ratio: Revisiting the Original Methods of Calculation. Meas. Eval. Couns. Dev..

[38] Polit D.F., Beck C.T. (2006). The Content Validity Index: Are You Sure You Know What’s Being Reported? Critique and Recommendations. Res. Nurs. Health.

[39] Zamanzadeh V., Ghahramanian A., Rassouli M., Abbaszadeh A., Alavi-Majd H., Nikanfar A.-R. (2015). Design and Implementation Content Validity Study: Development of an Instrument for Measuring Patient-Centered Communication. J. Caring Sci..

[40] Li C., Meng Z.X., Lin Y.B., Zhang L. (2025). Cross-Cultural Adaptation and Psychometric Evaluation of the Chinese Version of the Sickness Presenteeism Scale–Nurse (C-SPS-N): A Cross-Sectional Study. BMC Nurs..

[41] Crawford A.V., Green S.B., Levy R., Lo W.J., Scott L., Svetina D., Thompson M. (2010). Evaluation of Parallel Analysis Methods for Determining the Number of Factors. Educ. Psychol. Meas..

[42] Wood N.D., Akloubou Gnonhosou D.C., Bowling J. (2015). Combining Parallel and Exploratory Factor Analysis in Identifying Relationship Scales in Secondary Data. Marriage Fam. Rev..

[43] Li C.H. (2016). Confirmatory Factor Analysis with Ordinal Data: Comparing Robust Maximum Likelihood and Diagonally Weighted Least Squares. Behav. Res. Methods.

[44] Hu L.T., Bentler P.M. (1999). Cutoff Criteria for Fit Indexes in Covariance Structure Analysis: Conventional Criteria versus New Alternatives. Struct. Equ. Model..

[45] Kline R.B. (2016). Principles and Practice of Structural Equation Modeling.

[46] Rodriguez A., Reise S.P., Haviland M.G. (2016). Evaluating Bifactor Models: Calculating and Interpreting Statistical Indices. Psychol. Methods.

[47] Fornell C., Larcker D.F. (1981). Evaluating Structural Equation Models with Unobservable Variables and Measurement Error. J. Mark. Res..

[48] Liao Y., Mohd Hairon S., Yaacob N.M., Tengku Ismail T.A., Luo L. (2025). Psychometric Validation of a Knowledge, Attitudes, and Practices (KAP) Scale for Breast Cancer Screening among Chinese Women. BMC Public Health.

[49] Koo T.K., Li M.Y. (2016). A Guideline of Selecting and Reporting Intraclass Correlation Coefficients for Reliability Research. J. Chiropr. Med..

[50] Cheung G.W., Rensvold R.B. (2002). Evaluating Goodness-of-Fit Indexes for Testing Measurement Invariance. Struct. Equ. Model..

[51] Chen F.F. (2007). Sensitivity of Goodness of Fit Indexes to Lack of Measurement Invariance. Struct. Equ. Model..

[52] Muthén L.K., Muthén B.O. (2002). How to Use a Monte Carlo Study to Decide on Sample Size and Determine Power. Struct. Equ. Model..

[53] Magon A., Caruso R., Belloni S., Maga G., Conte G., Arrigoni C. (2025). Structural Equation Modeling and Monte Carlo Simulation in Clinical and Nursing Research: Insights into Sample Size, Opportunities, and Challenges. Epidemiol. Biostat. Public Health.

[54] Munuo A.E., Mugendi B.W., Kisanga O.A., Otieno G. (2016). Nutrition Knowledge, Attitudes and Practices among Healthcare Workers in Management of Chronic Kidney Diseases in Selected Hospitals in Dar es Salaam, Tanzania: A Cross-Sectional Study. BMC Nutr..

[55] Pu S., Peng H., Li Y., Huang X., Shi Y., Song C. (2024). Development of Standardized Nursing Terminology for the Process Documentation of Patients with Chronic Kidney Disease. Front. Nutr..

[56] Li Z., Zhen T., Zhao Y., Zhang J. (2023). Development and Assessment of a Nutrition Literacy Scale for Patients with End-Stage Kidney Disease Undergoing Dialysis and Its Correlation with Quality of Life. Ren. Fail..

[57] Irajpour A., Hashemi M.S., Abazari P., Shahidi S. (2024). The Effects of Peer Education on Treatment Adherence among Patients Receiving Hemodialysis: A Randomized Controlled Trial. Iran. J. Nurs. Midwifery Res..

[58] Zhianfar L., Nadrian H., Shaghaghi A. (2024). A Benchmarking and Evidence-Informed Gap Analysis of the Hemodialysis Care Provision in Iran. BMC Health Serv. Res..

[59] Fukada M. (2018). Nursing Competency: Definition, Structure and Development. Yonago Acta Med..

[60] Cowan D.T., Norman I., Coopamah V.P. (2007). Competence in Nursing Practice: A Controversial Concept—A Focused Review of Literature. Accid. Emerg. Nurs..

[61] Scott-Marshall H.K. (2024). Safe Limits on Work Hours for the Nursing Profession: A Rapid Evidence Review. Front. Glob. Womens Health.

[62] Pinto A.C.P., Tavares M.A.S., Pinto R.A.S.C., Pereira R.P.G. (2023). Attitudes and Barriers to Evidence-Based Nursing in Hemodialysis. Rev. Enferm. Referência.

[63] McDonald R.P. (1999). Test Theory: A Unified Treatment.

[64] Dunn T.J., Baguley T., Brunsden V. (2014). From Alpha to Omega: A Practical Solution to the Pervasive Problem of Internal Consistency Estimation. Br. J. Psychol..

[65] Mancin S., Palomares S.M., Sguanci M., Palmisano A., Gazineo D., Parozzi M., Ricco M., Savini S., Ferrara G., Anastasi G. (2025). Relational Skills of Nephrology and Dialysis Nurses in Clinical Care Settings: A Scoping Review and Stakeholder Consultation. Nurse Educ. Pract..

[66] Tang Y., Wen X., Tang X., Li X., Zhang L., Duan S., Long P., Zhou Z. (2024). Nutritional Nursing Competence of Clinical Nurses and Its Influencing Factors: A Cross-Sectional Study. Front. Nutr..

[67] Dellafiore F., Caruso R., Arrigoni C., Magon A., Baroni I., Alotto G., Quaccini C., Bianchi M., Bonetti L. (2021). The Development of a Self-Efficacy Scale for Nurses to Assess the Nutritional Care of Older Adults: A Multi-Phase Study. Clin. Nutr..

[68] Peng Y., Tan L., Zhang K., Zhu N., Dong H., Gao H. (2024). The Mediating Role of Nutritional Care Literacy on the Relationship between Self-Directed Learning Ability and Nursing Competence. BMC Nurs..

[69] Jackson H.S., MacLaughlin H.L., Vidal-Diez A., Banerjee D. (2019). A New Renal Inpatient Nutrition Screening Tool (Renal iNUT): A Multicenter Validation Study. Clin. Nutr..

[70] DuBois S., Spencer A., Nava A., Kaminski M., Gonzalez A.L., Arensberg M.B. (2025). Nourish the Mind: The Need for Nutrition-Focused Education in Nursing to Improve Health Outcomes. J. Prof. Nurs..

[71] Mancin S., Bragaglia F., Andreoli D., Morales Palomares S., Cangelosi G., Sguanci M., Bedin M., Godino L., Fabbri C., Gazineo D. (2024). Nursing Care and Postgraduate Education of Nephrology and Dialysis Nurses in Italy. G. Ital. Nefrol..

[72] Andreoli D., Morales Palomares S., Mancin S., Parozzi M., Gazineo D., Palmisano A., Angileri S.A., Ricco M., Anastasi G., Savini S. (2025). Exploring the Competencies of Nephrology Nurses: A Comprehensive Scoping Review. Int. Nurs. Rev..

