# Supplementary material for: Questionnaire on Nursing Competencies in Nutritional Care for Chronic Kidney Patients: Development and Validation
Source: Nurs Rep. 2026 Feb 24;16(3):78. doi: 10.3390/nursrep16030078 (PMC13029465; doi:10.3390/nursrep16030078)
Supplement: Supplementary file 1 [file nursrep-16-00078-s001.zip › nursrep-4067962-supplementary.pdf]

Formazione e Competenze Infermieristiche nella
Nutrizione dei Pazienti con MRC in ESRD

I PARTE - COMPETENZE SPECIFICHE

Per cortesia, leggi attentamente ciascuna affermazione e indica il tuo grado di accordo utilizzando la seguente scala Likert:
1 = Fortemente in disaccordo 2 = In disaccordo 3 = Né d'accordo né in disaccordo
4 = D'accordo 5 = Fortemente d'accordo

1. **Ritieni che, i seguenti interventi assistenziali nutrizionali** nei pazienti con Malattia Renale Cronica in ESRD **DOVREBBERO ESSERE EFFETTUATI nel contesto clinico in cui lavori:**

1 a. Monitorare l'apporto di energia e nutrienti

1	2	3	4	5

1 b. Individuare la presenza di malnutrizione attraverso criteri validati (ad es. ESPEN GLIM), in collaborazione con il medico nefrologo

1	2	3	4	5

1 c. Collaborare con altri professionisti sanitari (ad es. nutrizionista/dietista, nefrologo, psicologo ed assistente sociale) per la gestione nutrizionale

1	2	3	4	5

1 d. Educare alla corretta gestione nutrizionale in collaborazione con il dietista/nutrizionista, in base alle condizioni cliniche

1	2	3	4	5

1 e. Valutare l'apporto di sodio, potassio e fosforo

1	2	3	4	5

1 f. Valutare lo stato di idratazione

1	2	3	4	5

1 g. Indagare periodicamente la presenza di alterazioni di gusto e olfatto

1	2	3	4	5

1 h. Identificare e gestire le complicanze nutrizionali (iperkaliemia, ipocalcemia e iperfosfatemia)

1	2	3	4	5

1 i. Applicare le Linee Guida nutrizionali

1	2	3	4	5

1 l. Altro (specificare):

---

2. Ritieni che, nel tuo contesto clinico, gli infermieri **SIANO IN GRADO DI EFFETTUARE** i seguenti interventi assistenziali nutrizionali nei pazienti con Malattia Renale Cronica in ESRD:

2 a. Monitorare l’apporto di energia e nutrienti

1	2	3	4	5

2 b. Individuare la presenza di malnutrizione attraverso criteri validati (ad es. ESPEN GLIM), in collaborazione con il medico nefrologo

1	2	3	4	5

2 c. Collaborare con altri professionisti sanitari (ad es. nutrizionista/dietista, nefrologo, psicologo ed assistente sociale) per la gestione nutrizionale

1	2	3	4	5

2 d. Educare alla corretta gestione nutrizionale in collaborazione con il dietista/nutrizionista, in base alle condizioni cliniche.

1	2	3	4	5

2 **e**. Valutare l’apporto di sodio, potassio e fosforo

1	2	3	4	5

2 **f**. Valutare lo stato di idratazione

1	2	3	4	5

2 **g**. Indagare periodicamente la presenza di alterazioni di gusto e olfatto

1	2	3	4	5

2 **h**. Identificare e gestire le complicanze nutrizionali (iperkaliemia, ipocalcemia e iperfosfatemia)

1	2	3	4	5

2 **i**. Applicare le Linee Guida nutrizionali

1	2	3	4	5

2 **l**. Altro (specificare): ______________________________

3. **Ritieni che, i seguenti interventi assistenziali** nutrizionali nei pazienti con Malattia Renale Cronica in ESRD vengano **EFFETTUATI CON FREQUENZA nel tuo contesto clinico:**

3 **a**. Monitorare l’apporto di energia e nutrienti

1	2	3	4	5

3 **b**. Individuare la presenza di malnutrizione attraverso criteri validati (ad es. ESPEN GLIM), in collaborazione con il medico nefrologo

1	2	3	4	5

3 **c**. Collaborare con altri professionisti sanitari (ad es. nutrizionista/dietista, nefrologo, psicologo ed assistente sociale) per la gestione nutrizionale

1	2	3	4	5

3 **d.** Educare alla corretta gestione nutrizionale in collaborazione con il dietista/nutrizionista, in base alle condizioni cliniche

1	2	3	4	5

3 **e**. Valutare l'apporto di sodio, potassio e fosforo

1	2	3	4	5

3 **f**. Valutare lo stato di idratazione

1	2	3	4	5

3 **g.** Indagare periodicamente la presenza di alterazioni di gusto e olfatto

1	2	3	4	5

3 **h**. Identificare e gestire le complicanze nutrizionali (iperkaliemia, ipocalcemia e iperfosfatemia)

1	2	3	4	5

3 **i**. Applicare le Linee Guida nutrizionali

1	2	3	4	5

3 **l.** Altro (specificare):
